# Application of cell-sheet engineering for new formation of cementum around dental implants

**DOI:** 10.1016/j.heliyon.2019.e01991

**Published:** 2019-06-29

**Authors:** Kengo Iwasaki, Kaoru Washio, Walter Meinzer, Yuka Tsumanuma, Kosei Yano, Isao Ishikawa

**Affiliations:** a Institute of Dental Research, Osaka Dental University, Japan; b Institute of Advanced Biomedical Engineering and Science, Tokyo Women's Medical University, Japan; c Department of Periodontology, Graduate School of Medical and Dental Sciences, Tokyo Medical and Dental University (TMDU), Japan

**Keywords:** Biomedical engineering, Dentistry, Bioengineering, Cell biology, Periodontal ligament, Stem cells, Dental implant., Cementum, Cell sheet engineering

## Abstract

Periodontal disease involves the chronic inflammation of tooth supporting periodontal tissues. As the disease progresses, it manifests destruction of periodontal tissues and eventual tooth loss. The regeneration of lost periodontal tissue has been one of the most important subjects in periodontal research. Since their discovery, periodontal ligament stem cells (PDLSCs), have been transplanted into periodontal bony defects to examine their regenerative potential. Periodontal defects were successfully regenerated using PDLSC sheets, which were fabricated by cell sheet engineering in animal models, and for which clinical human trials are underway. To expand the utility of PDLSC sheet, we attempted to construct periodontal tissues around titanium implants with the goal of facilitating the prevention of peri-implantitis. In so doing, we found newly formed cementum-periodontal ligament (PDL) structures on the implant surface. In this mini review, we summarize the literature regarding cell-based periodontal regeneration using PDLSCs, as well as previous trials aimed at forming periodontal tissues around dental implants. Moreover, the recent findings in cementogenesis are reviewed from the perspective of the formation of further stable periodontal attachment structure on dental implant. This mini review aims to summarize the current status of the creation of novel periodontal tissue-bearing dental implants, and to consider its future direction.

## 1 Introduction

Periodontal disease is one of the most widespread infectious/inflammatory diseases in humans that is caused mainly by the presence of gram-negative bacteria. The disease is characterized by chronic inflammation and destruction of the tooth-supporting periodontal tissues [1, 2]. If the disease is not properly treated, and disease progression is not arrested, the tooth may require extraction. Therefore, the regeneration of periodontal tissues has been an important and dynamic field of periodontal research. It has been demonstrated that the periodontal ligament (PDL) – a thin connective tissue that bridges tooth and bone – plays a key role in periodontal tissue regeneration by being a source of cells having regenerative potential for periodontal tissues [3, 4, 5, 6]. A unique PDL cell population has been identified that exhibits mesenchymal stem cell (MSC)-like characteristics. Named periodontal ligament stem cells (PDLSCs) in 2004 [7], PDLSCs demonstrated the capacity to differentiate into three mesenchymal lineages of cells *in vitro*; osteoblasts, adipocytes, and chondrocytes [7, 8, 9]. In addition to its MSC-like characteristics, PDLSCs were found to differentiate into cementoblasts and form cementum-PDL like structures upon transplantation *in vivo* [7, 10]. Additionally, PDLSCs exhibited the marker expression of pericytes, a mesenchymal-like cell type localized around blood vessels. This finding is in keeping with the classical concept that stem/progenitor cells are located in the perivascular niche in PDL [11]. Thus, PDLSCs are now thought to be putative adult stem cells in PDL.

Dental implants are among the most promising treatment options available following exodontia [12]. Implants prepared from titanium integrate directly with the bone (osseointegration) and can function stably in the oral cavity. However, dental implants lack important functional components associated with natural teeth, such as cementum and PDL, and thus buffering of mastication force, as well as immunological defense systems, would not be expected with implants.

Recent advances, in tissue engineering and stem cell biology, have made it possible to regenerate new tissues by transplanting *ex vivo* expanded cultured stem cells. We recently succeeded in forming a cementum-PDL structure on a titanium dental implant surface [13] and we suggested a novel implant system that bears surrounding periodontal tissues. In this mini-review, we summarize previous trials regarding formation of periodontal tissues on titanium surface. Furthermore, we also summarize studies regarding the induction of cementum, the key tissue facilitating stable attachment between an implant and periodontal tissues.

## 2 Main text

### 2.1 Periodontal regeneration by PDLSCs cell sheet

MSCs are the most widely investigated cell type for cell-based treatment because of their characteristics such as multi-differentiation capacity, immunomodulation, anti-apoptosis, angiogenesis, and cell recruitment [14]. Besides these MSC characteristics, PDLSCs possess a unique potential to form cementum, and this characteristic of PDLSCs has stimulated many researchers to examine periodontal regeneration by transplantation of PDLSCs. We have made significant contributions to this research field in periodontology, by demonstrating the regeneration of periodontal tissues after transplantation of a “PDLSC sheet” prepared using cell sheet engineering in various animal models since 2005 [15,16]. Cell sheet engineering is a unique tissue engineering method to obtain cells in a sheet format, which allows collection of the cell sheet without destruction of extracellular matrix components secreted from cells. We have previously reported on human clinical studies using PDL cell sheet transplantation for periodontal regeneration in Japan and some cases have been published [17].

The understanding that transplantation of PDLSCs induces regeneration of periodontal tissues, is gaining wide acceptance and review papers have summarized the results of PDLSC transplantation studies [18, 19, 20]. PDLSC transplantation is now considered one of the promising regenerative approaches for periodontal tissues.

### 2.2 Construction of dental implant bearing periodontal tissues by PDLSCs

While the initial results using PDLSC sheets for periodontal regeneration appear promising, a new trial is underway to extend the applicability of this powerful tool to develop periodontal tissues on the surface of titanium implants. It is a novel tissue engineering approach to form periodontal tissue-bearing implants.

Dental titanium implants directly integrate with the bone and lack several important functional structures associated with a natural tooth, such as cementum and PDL. These attachment structures, normally associated with teeth, could function collectively as a buffering structure against the forces of mastication to help protect the implant from traumatic mechanical load. Moreover, the direct interface between oral epithelium and titanium implant presents higher risks of bacterial invasion and consequential inflammatory peri-implant mucositis and peri-implantitis. Recent studies showed that peri-implantitis is considered a major and growing problem in dentistry [21]. Since the PDL function as a reservoir of immune cells, the formation of a cementum-PDL structure around dental implants could help protect the implant-bone interface from oral bacterial challenge.

To overcome the above-mentioned shortcomings of dental implants, successful formation of periodontal tissues has recently been demonstrated on titanium implants after transplantation of PDLSC sheets in rats and dogs [13]. Histological evidence of periodontal tissue formation on dental implants has been reported previously as summarized in Table 1 [22, 23, 24, 25, 26, 27, 28, 29, 30, 31, 32, 33, 34, 35, 36]. As a search strategy and selection criteria, data for this table were collected by a search using PubMed and MEDLINE. Search terms included “implant”, “periodontal ligament”, “cementum”, “periodontal tissues”, and “titanium”. English articles from 1990 to April, 2019 were referenced. The first report regarding PDL formation on titanium implants was published by Buser *et al.* in 1990 [22]. They unexpectedly found the formation of PDL on the surface of dental implants located close to retained tooth roots in monkeys. Then, Choi *et al.* demonstrated the formation of cementum-PDL like tissue by transplanting a titanium implant with PDL cells cultured on its surface [25]. These findings suggested the possibility of periodontal tissue formation on dental implant surfaces; however, the methodology was not yet established. Additionally, some studies of a unique implant placement method for avoiding alveolar ridge atrophy (socket shield technique [SST]), demonstrated new formation of cementum and PDL tissue on titanium implant [31, 36]. Hürzeler *et al.* found the formation of cementum-PDL on implant surfaces that were placed beside retained root fragments in dogs [31]. However, Schwimer *et al.* reported that no PDL formation was observed on the surface of a failed implant recovered from a human subject 2 years after SST treatment [36]. The type of periodontal tissue formation on dental implants with SST remains controversial.

**Table 1:** Cementum-PDL formation on implant.

Cell sourse	Method of cell transplantation	Type of Implant	Implant surface modification	New cementum-PDL like tissue	Experimental animal	Healing period	Year	Reference
Retained tooth root	-	Hollow-cylinder Ti implant	Plasma-sprayed	Yes	Monkey	12 months	1990	# [22]
Retained tooth root	-	Self-tapping screw tiype Ti implant	-	Yes	Monkey	3 months	1993	# [23]
Tooth root	-	Screw type Ti implant	HA	Yes	Dog	3 months	2000	# [24]
PDL cells	Direct cell culture on implant	Screw type Ti implant	-	Yes	Dog	3 months	2000	# [25]
Retained tooth root	-	-	-	only cementum	Human (case report)	1 year	2002	# [26]
PDL tissue	-	Self-tapping cylinder type Ti implant	Plasma sprayed, Sandblasting Large-grit acid-etching	Yes	Dog	4 months	2005	# [27]
Retained PDL in extracted socket	-	Custam made Ti implant	-	Yes	Rat	4 weeks	2005	# [28]
Contacted tooth with Implant by orthodontic movement	-	Ti implant	HA	Yes	Dog	8 weeks	2005	# [29]
PDL cells	Direct cell culture on implant in bioreactor	Cone-shaped cylinder Ti pin	HA	Yes	Dog	20 weeks	2010	# [30]
Retained root fragment in contact with implant (SST)	-	Ti implant	-	Yes	Dog	4 months	2010	# [31]
PDL cells	Matrigel as a scafold	Custam made Ti implant	Sandblasting Large-grit acid-etching	Yes	Rat	12 weeks	2011	# [32]
Remained PDL in extracted socket	-	Custam made Ti implant	HA	only PDL	Rat	4 weeks	2012	# [33]
Dental follicle taken from tooth germ	Wrapping of implant with dental follicle	Custam made Ti implant	HA	Yes	Mouse	30 days	2014	# [34]
PDL cells + HUVEC + cementoblasts + ERM cells	Cell Sheet	Custam made Ti implant	HA	Yes	Mouse	8 weeks	2017	# [35]
PDL cells	Cell Sheet	Cylinder type Ti implant	Acid etching, blasting, calcium phosphate coating	Yes	Dog	11 weeks	2018	# [13]
Retained root fragment in contact with implant (SST)	-	Ti implant	-	No	Human (Failed implant)	About 2 years	2018	# [36]

PDL: periodontal ligament, HUVEC: human umbilical vein endothelial cell, ERM: epithelial cell rests of Malassez, HA: hydroxyapatite, Ti: titanium, SST: Socket Shield Technique.

**Fig. 1:**
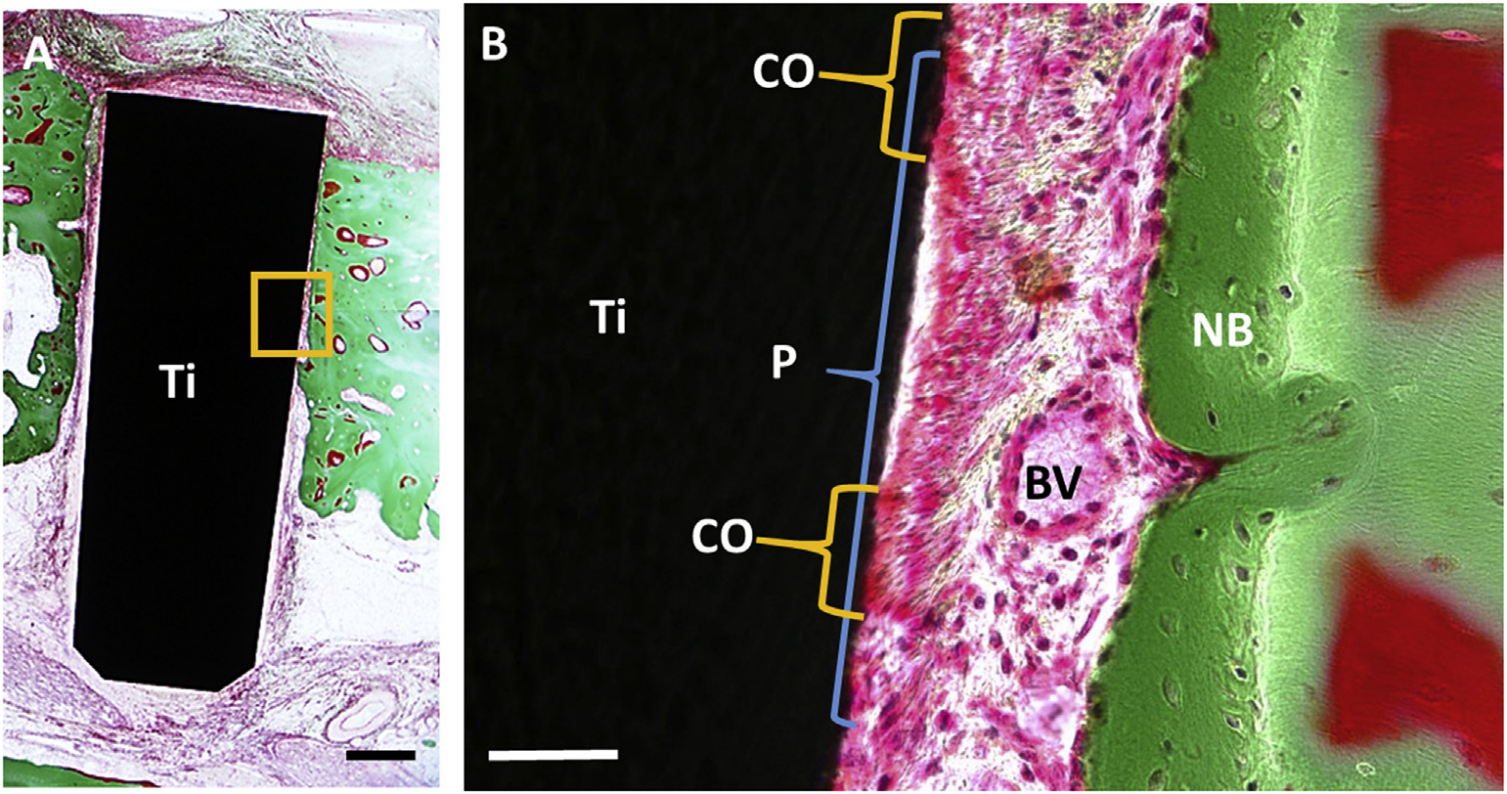
Periodontal tissue formation around titanium implant in dog. Histological images of titanium implant transplanted with PDLSC sheet in bone defect of dog mandible at 11 weeks post-implantation. Image of implant surface at lower magnification is demonstrated in A and magnified image of boxed area is shown in B. Newly formed PDL-like structure was observed on titanium surface. In PDL-like space observed between titanium implant and bone, running of abundant collagen bundles are prominent. On the implant surface, cementoid-like structure is also observed. Ti: titanium implant, CO:cementoid like tissue, P:PDL-like tissue, BV: blood vessel, NB: new bone. Figure 6A and B from Washio K *et al.* “In vivo Periodontium Formation Around Titanium Implants Using Periodontal Ligament Cell Sheet”, published in Tissue Engineering Part A 2018. Volume 24, pp.1273–1282 with permission from Mary Ann Liebert, inc., New Rochell, NY.

Applying “cell sheet engineering”, we examined periodontal tissue formation on titanium dental implants and demonstrated successful establishment of periodontal tissues [13]. We prepared PDL cell sheets using temperature-responsive cell culture dishes and transplanted them with dental implants into a canine bone defect model. Eleven weeks post-transplantation, histological sections showed PDL-cementum-like structures on the titanium surface (Fig. 1). For this study, we modified the implant surface with acid etching, blasting, and calcium phosphate (CaP) coating to facilitate cell attachment and proliferation. Our results suggested that the transplantation of PDLSC sheets constructed using cell sheet engineering is a useful method for the formation of periodontal tissues around titanium implants.

### 2.3 Periodontal tissue formation around implants and cementogenesis

As mentioned earlier, the possibility of periodontal tissue formation around titanium implants has been suggested. However, there is some room for improvements in the methodology. Detachment of collagen fibers from the titanium surface and the parallel orientation of PDL collagen fibers relative to the titanium surface have been observed in animal studies. Thus, it is important to improve the implant/PDL interface in the interest of stability of the PDL-implant association, and the robust formation of cementum on the implant surface may be the key to improving the construction of periodontal tissues around implants.

Since the induction of cementum formation on the dental implant is vital to the construction of the PDL-implant, it is essential to have an understanding of the mechanisms of cementogenesis. Unfortunately, the available information regarding the induction of cementum formation is limited. Previously, the essential cellular signals for cementum formation during tooth root formation have been examined in genetically-targeted mice. Among the various molecules tested, Wnt was the most prominent signaling molecule that showed phenotypic changes in root cementum. Lim *et al.* have reported thick cementum formation in mice upon deletion of Wnt signaling, specifically in odontoblasts and osteoblasts [37]. Furthermore, Kim *et al.* have demonstrated that mice with stabilized Wnt signaling, specifically in dental mesenchymal cells, exhibit thick cementum [38]. Additionally, Nemoto *et al.* have reported that Hertwig's epithelial root sheath and epithelial cell rests of Malassez, important regulators of cementogenesis and homeostasis, produced Wnt3a [39]. These results strongly suggested that Wnt signaling facilitates the formation of cementum *in vivo*.

The research group led by Sommerman *et al.* have demonstrated that acellular cementum formation is sensitive to local inorganic phosphate (Pi)/inorganic pyrophosphate (PPi) concentration in their series of studies using knockout mice, including the deletion of progressive ankylosis gene (Ank) [40], tissue non-specific alkaline phosphatase (TN-ALP) [41], ectonucleotide pyrophosphatase/phosphodiesterase 1 (ENNP1) [41], and integrin binding sialoprotein [42]. All of these molecules control the concentrations of Pi and PPi. Pi has an important role in mineralization through hydroxyapatite crystal growth, while PPi competitively inhibits hydroxyapatite precipitation. Thus, the local Pi/PPi level controls the mineralization of hard tissues. Acellular cementum formation was increased in a higher Pi/PPi environment and this increment was observed specifically in cementum. Their results demonstrated that the higher Pi/PPi ratio may be an induction signal of cementum formation and suggested the possibility of artificial formation of cementum through controlling the local level of Pi/PPi.

Cementoblasts and osteoblasts share various hard tissue-forming characteristics, such as mineralized nodule formation, osteoblast differentiation marker expression, and higher alkaline phosphatase activity [43]. However, specific induction signals and expression markers for cementoblasts have not been identified yet. This insufficient information makes the investigation of cementoblasts difficult *in vitro*. Thus far, only cementum attachment protein (CAP) and Cementum protein-1 (CEMP-1) have been identified as putative markers of cementoblasts [44, 45]. Various studies have investigated the mechanisms of cementoblast differentiation using PDL cells. Komaki *et al.* have demonstrated that alkaline phosphatase-positive cells in cultured PDL cells expressed higher levels of CEMP-1. They also found that bone morphogenetic protein 2, potent inducer of osteoblast differentiation, decreased CEMP-1 expression [46]. Moreover, Gauthier et al. demonstrated the reduction of CEMP-1 and CAP expression after osteoblastic differentiation of PDLSCs, and CEMP-1 expression enhancement with the addition of ascorbic acid to PDLSCs [47]. These results suggest differences in *in vitro* induction signaling for osteoblasts and cementoblasts, although they share various characteristics of hard tissue forming cells.

The interface between cementum and titanium surfaces is arguably the most important component essential for the clinical stability of dental implants with periodontal tissues. Therefore, an improved understanding of cementoblast differentiation mechanisms and appropriate induction methods for cementoblasts is critical, and needs to be pursued with further detailed investigations both *in vivo* and *in vitro*. Furthermore, artificial induction of cementum formation is not only useful for the PDL-implant, but also important for periodontal regeneration involving natural teeth. The further investigation of cementum forming mechanisms will have significant benefits for both functional implants and periodontal treatment.

### 2.4 Outstanding questions

Construction of periodontal tissues around implants may be beneficial for the improved longevity of dental implants, because the PDL contributes physiological protective functions including immune surveillance and buffering of large occlusal forces. However, several questions remain unanswered relative to the current status of implants with associated periodontal tissues. Although a cementum-PDL structure has been formed on an implant surface, it is unclear to what extent it works functionally. Buffering of mechanical force by a PDL is advantageous for the reduction of occlusal force applied to an implant; however, the attachment strength at the interface between cementum and implant is as yet unknown. Moreover, another question is whether titanium is the best material for a functional tooth substitute. Historically, titanium has been the implant material of choice, because its unique characteristics allow for a rigid connection to bone. However, when it comes to selecting an implant material around which to construct periodontal tissues, titanium may not always be the best choice. Therefore, the selection of a suitable material for the PDL-implant is remains to be determined and is important topic for future research.

## 3 Conclusion

Since its discovery, the potential of PDLSCs to regenerate periodontal tissue has been widely investigated. Most results demonstrated periodontal regeneration by PDLSCs in various experimental settings. Among them, “cell sheet engineering” is one of the most successful methods for the regeneration of periodontal tissues by transplantation of PDLSCs. Additionally; the potential of PDLSC sheets to form periodontal tissues opens the door to experimental trials that could provide a new concept in dental treatment: implants with periodontal tissues. Although the realization of such treatment has not yet been achieved, the further development of this tissue engineering approach is expected to provide a novel dental treatment option in the future.

## Declarations

### Author contribution statement

All authors listed have significantly contributed to the development and the writing of this article.

### Funding statement

This work was supported by the Creation of Innovation Centers for Advanced Interdisciplinary Research Areas Program, in the Project for Developing Innovation Systems “Cell Sheet Tissue Engineering Center (CSTEC),” from the Ministry of Education, Culture, Sports, Science, and Technology (MEXT), Japan, MEXT/JSPS KAKENHI Grant Number JP 26861687. Dental corporation Tokushin-Kai group also supported this study financially.

### Competing interest statement

The authors declare the following conflict of interests: I.I. is an adviser of Dental Corporation Tokushin-Kai group and reports personal fees during the conduct of the study. In addition, I.I. has a patent US20170157292A1, EP3162385A4, JPWO2015199245A1 pending. The remaining authors have no conflicts of interest to declare.

### Additional information

No additional information is available for this paper.
